# Imaging probes for non-invasive tumoral detection and functional monitoring of cancer multidrug resistance

**DOI:** 10.20517/cdr.2019.86

**Published:** 2020-02-20

**Authors:** Filipa Mendes, Lurdes Gano, Jorge Grilo, Susana Cunha, Célia Fernandes, António Paulo

**Affiliations:** ^1^DECN- Department of Nuclear Engineering and Sciences and C^2^TN - Center for Nuclear Sciences and Technologies, Instituto Superior Técnico, Universidade de Lisboa, Estrada Nacional 10, Bobadela LRS 2695-066, Portugal.; ^2^(Present adress) iMed. Ulisboa - Research Institute for Medicines, Faculdade de Farmácia, Universidade de Lisboa, Av. Professor Gama Pinto, Lisboa 1649-003, Portugal.; ^#^Authors contributed equally.

**Keywords:** Noninvasive imaging, imaging probes, tumoral detection, multiple drug resistance, P-glycoprotein

## Abstract

**Aim:** Several cationic radiotracers originally developed as myocardial perfusion agents have shown potential for both early detection of cancer and non-invasive monitoring of multiple drug resistance (MDR) by single photon emission computed tomography. We have introduced two cationic complexes, ^99m^Tc-DMEOP [di-methoxy-tris-pyrazolyl-^99m^Tc-(CO)_3_] and ^99m^Tc-TMEOP [tri-methoxy-tris-pyrazolyl-^99m^Tc-(CO)_3_], which showed excellent preclinical results as cardiac imaging probes, namely a persistent heart uptake with rapid blood and liver clearance. This study aimed at the evaluation of their usefulness for tumoral detection and functional assessment of MDR.

**Methods:** The uptake and efflux kinetics of ^99m^Tc-DMEOP and ^99m^Tc-TMEOP were evaluated in human prostate, lung, and breast cancer cell lines, including drug-resistant cell lines that are known to overexpress the MDR P-glycoprotein (Pgp). The effects of MDR modulators were also studied. *In vivo* studies were performed in xenografted tumor models, and the MDR phenotype of the tumors was confirmed by Western blot.

**Results:** The uptake kinetics of both complexes in human cancer cell lines is comparable with the one found for ^99m^Tc-Sestamibi, increasing over time. The uptake of ^99m^Tc-TMEOP is greatly reduced in cells overexpressing Pgp and increased in the presence of a Pgp modulator. In nude mice, the tumor uptake of ^99m^Tc-TMEOP was higher in the MCF-7 xenografts compared with the MCF7 Pgp tumors.

**Conclusion:** The uptake kinetics of both complexes in human cancer cell lines is comparable with the one found for ^99m^Tc-Sestamibi, increasing over time. The uptake of ^99m^Tc-TMEOP is greatly reduced in cells overexpressing Pgp, and increased in the presence of a Pgp modulator. In nude mice, the tumor uptake of ^99m^Tc-TMEOP was higher in the MCF-7 xenografts compared with the MCF7 Pgp tumors.

## Introduction

Cancer is a leading cause of death worldwide. The World Health Organization reported that cancer accounted for 9.6 million deaths (around 17% of all deaths worldwide) in 2018^[1]^.

A major obstacle to successful cancer chemotherapy is drug resistance^[2,3]^. Many tumors are intrinsically resistant to chemotherapy (e.g., kidney, pancreas, liver, and colon), whereas others initially respond to treatment, but acquire resistance to selected cytotoxic drugs during chemotherapy. One form of resistance, so-called multidrug resistance (MDR), is responsible for the failure of tumors to respond to a wide spectrum of chemotherapeutic agents^[2,3]^.

Numerous mechanisms have been proposed to mediate intrinsic or acquired MDR in cancer cells^[4]^. MDR can result from non-cellular mechanisms such as limited vascular accessibility or from cellular mechanisms including modification of the drug target, changes in ability to repair DNA following drug-induced damage, disruptions in apoptotic pathways, and changes in the expression of proteins and enzymes associated with tumor resistance. To date, the most widely studied cellular mechanisms of tumor resistance are those associated with drug efflux mechanisms involving members of the adenosine triphosphate (ATP)-binding cassette (ABC) membrane transporter family, most importantly P-glycoprotein (Pgp), but also multidrug-resistant protein 1 (MRP1) and homologs (MRP2-6) and breast cancer resistance protein (BCRP). These transporters are found in normal cells, where their role has been identified as one of protection against, and clearance of, excessive extracellular and intracellular concentrations of xenobiotics and toxins. An overarching feature of ABC transporter-expressing tumor cells is their reduced ability to accumulate certain cytotoxic agents intracellularly, resulting in ineffective cellular levels that fail to bring about cell death^[3,4]^.

Measurement of MDR is one potentially important marker in planning systemic therapy, as accurate selection of chemosensitive patients would result not only in effective treatment of patients and avoidance of potentially toxic side effects but also in significant cost savings for health care providers without a significant loss of life expectancy for patients^[5,6]^.

The methodologies for the determination of mRNA and protein levels are important for assessing Pgp expression, but they have poor sensitivity and specificity and fail to provide functional information. Therefore, methods for functionally interrogating Pgp, MRP1, and BCRP transport activity have been sought, as this type of phenotypic evaluation is very important to avoid ineffective and potentially toxic treatments^[5]^. Significant effort has been directed toward the non-invasive detection of transporter-mediated resistance using radiopharmaceuticals characterized as transport substrates for Pgp and other MDR proteins (for review, see^[7,8]^).

Despite of an incomplete understanding of the transport mechanisms, several lipophilic cations originally developed for myocardial perfusion SPECT imaging were identified as a class of compounds which accumulate in tumor cells due to the increased negative mitochondrial potentials and that act as substrates for Pgp and MRP1^[5]^. ^99m^Tc-Sestamibi was the first radiopharmaceutical recognized as a useful probe for cancer early detection and functional Pgp imaging transport^[9]^, followed by other ^99m^Tc complexes, namely ^99m^Tc-tetrofosmin^[10]^, ^99m^Tc-furifosmin^[11]^, and ^99m^Tc-areneisonitrile^[12]^, and, more recently, mixed-ligand ^99m^Tc-dithiocarbamate complexes^[13]^ and a ^99m^Tc-labeled triphenylphosphonium cation^[14]^.

Several studies have reported that absence or reduced uptake of ^99m^Tc-sestamibi may serve to predict poor response to therapy in several human tumors, including lymphoma^[15]^, nasopharyngeal cancer^[16]^, thyroid cancer^[17]^, lung cancer^[18]^, breast cancer^[19,20]^, ovarian cancer^[21]^, osteosarcoma^[22]^, and renal malignancies^[23]^. A meta-analysis showed that ^99m^Tc-Sestamibi could play a significant role in the management of lung cancer as it can predict accurately which patients will respond to chemotherapy^[6]^. However, diagnostic and prognostic values of ^99m^Tc-Sestamibi and ^99m^Tc-tetrofosmin are often limited due to their high uptake in the liver, which makes it very difficult to detect small lesions in the chest and abdominal regions. Thus, there is an unmet medical need for radiotracers that are able to monitor noninvasively the MDR transport function in tumors^[5,8,24]^.

**Figure 1:**
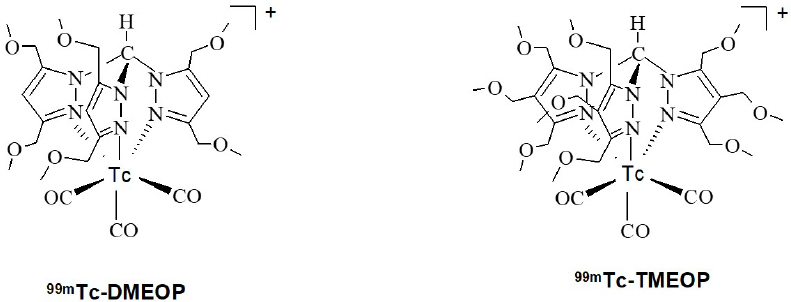
Structure of ^99m^Tc-DMEOP and ^99m^Tc-TMEOP complexes

Based on the tricarbonyl technology and using ^99m^Tc organometallic complexes as a lead structure, we have searched for better performing myocardial imaging agents^[25,26]^ and introduced and characterized a new family of cationic lipophilic ^99m^Tc tricarbonyl complexes. Based on promising pre-clinical studies, two of them, ^99m^Tc-TMEOP [tri-methoxy-tris-pyrazolyl-^99m^Tc-(CO)_3_] and ^99m^Tc-DMEOP [di-methoxy-tris-pyrazolyl-^99m^Tc-(CO)_3_], were considered good myocardial SPECT imaging agents, with a more favorable biological profile than the compounds in clinical use^[25-28]^ [Figure 1]. ^99m^Tc-TMEOP and ^99m^Tc-DMEOP showed very good results in the pre-clinical studies, exhibiting a good biological profile with rapid hepatic clearance. Significantly, ^99m^Tc-TMEOP reaches in an animal model a heart/liver ratio of 1 in about half the time of the compounds in clinical use (^99m^Tc-Sestamibi and ^99m^Tc-tetrofosmin), allowing for a better image quality. The evaluation of the heart uptake mechanism of ^99m^Tc-TMEOP showed that it is similar to that of the other reported monocationic ^99m^Tc cardiac agents, and is associated with its accumulation in the mitochondria^[27]^. We also performed *in vivo* studies to evaluate the effect of cyclosporine A, a Pgp inhibitor, on the biodistribution of ^99m^Tc-TMEOP in rats, and observed that this treatment induced a significant decrease in the washout rate from liver, kidneys, and lungs, organs with a high Pgp expression. This led us to propose that its fast liver and kidneys clearance kinetics is mediated by Pgp. Interestingly, the liver clearance of ^99m^Tc-TMEOP is faster than for ^99m^Tc-sestamibi and ^99m^Tc-tetrofosmin, thus suggesting a more efficient recognition by Pgp of ^99m^Tc-TMEOP and, consequently, a potentially enhanced ability of imaging the Pgp function associated to MDR tumors.

Given the better biological profile of ^99m^Tc-TMEOP and ^99m^Tc-DMEOP as myocardial imaging agents compared with the compounds in use, we foresee that they could have a better performance as tumoral detection and MDR imaging agents. Therefore, our aim with this work was to evaluate the ability of ^99m^Tc-TMEOP and ^99m^Tc-DMEOP to target cancer cells and to functionally assess MDR.

## Methods

### Synthesis of ^99m^Tc-DMEOP and ^99m^Tc-TMEOP

One milliliter of a [^99m^Tc(H_2_O)_3_(CO)_3_]^+^ solution (pH 4), obtained using an IsoLink® kit (Covidien, Petten, the Netherlands), ranging from 37 and 370 MBq, was added to a nitrogen purged vial containing 2.8 mg [Na(DMEOP)_2_]I or 3.4 mg of [Na(TMEOP)_2_]I, corresponding to a ligand concentration of 2.5 × 10^−3^ M. The solution was heated to 100 °C for 30 min. After cooling, the pH was adjusted to 7.4 using 0.1 M NaOH. ^99m^Tc-DMEOP and ^99m^Tc-TMEOP were analyzed by reverse-phase high performance liquid chromatography (RP-HPLC) using a Nucleosil C18 column (10 mm, 250 mm × 4 mm) and a gradient of 0.1% trifluoroacetic acid and CH_3_CN at a flow rate of 1 mL/min. ^99m^Tc-sestamibi (Cardiolite, Bristol-Myers-Squibb) and ^99m^Tc-tetrofosmin (Myoview, GE Healthcare) were prepared according to the manufacturer’s instructions.

### Cell lines

A panel of human tumoral cell lines was used: A2780 ovarian carcinoma (ECACC 93112519), A431 squamous vulvar carcinoma (ECACC 85090402), Hela cervix epitheloid carcinoma (ECACC 93021013), MDA MB 231 metastatic breast adenocarcinoma (ECACC 92020424), MCF7 metastatic breast adenocarcinoma (ECACC 86012803), H69 small cell lung carcinoma (ECACC 91091802), and PC3 prostate adenocarcinoma (ECACC 90112714).

In addition, the following drug-resistant variants were used: MCF7 Pgp (gift from D. Piwnica-Worms) and H69 Lx4 (ECACC 96042329), which overexpress Pgp, and H69 Ar (ATCC CRL-11351), which overexpresses MRP1. The MCF7 neo cell line (also a gift from D. Piwnica-Worms), which corresponds to MCF7 cells transfected with an empty vector, was used as a transfection control for MCF7 Pgp.

A431, Hela, MCF7, and MDA MB231 were cultured in Dulbecco’s Modified Eagle Medium (DMEM) with 10% fetal bovine serum (FBS), while A2780, H69, and PC3 were cultured in RPMI with 10% FBS (all from Gibco Life Technologies). MCF7 Pgp and MCF7 neo grew in DMEM with 10% FBS supplemented with 1 mg/mL of G418 (Sigma) and H69 Lx4 in RPMI with 10% FBS supplemented with 0.4 mg/mL of doxorubicin (Sigma). H69 AR were grown in RPMI with 20% FBS. Cells were cultured at 37 °C in a humidified atmosphere with 5% CO_2_ and regularly checked for mycoplasma contamination.

### Cell uptake and efflux studies

For the uptake studies, cells were cultured at a density of 2 × 10^5^ cells/well in 24-well plates. After 24 h, cells were incubated with medium containing either ^99m^Tc-DMEOP or ^99m^Tc-TMEOP at ≈ 37 kBq/mL. At selected time points, the incubation was stopped by washing twice with ice-cold phosphate buffer saline (PBS) solution and the cells were lysed with NaOH 1 M. The radioactivity in the total cellular extracts was measured using a gamma counter (LB 2111, Berthold) and is reported as proportion to the total applied radioactivity and normalized to 1 × 10^6^ cells. Each experiment was performed with four replicates and data are presented as average ± SEM of typically 3-4 independent experiments.

For efflux studies, the cells were incubated with the ^99m^Tc-complexes at 37 °C for 1 h. Thereafter, incubation was stopped, the growth medium removed, the cells washed with PBS, and fresh medium added. The radioactivity released at different time points after incubation was measured on a gamma counter as above, and is reported as proportion to the total radioactivity taken up by the cells after 1 h of incubation. Each experiment was performed with four technical replicates and data are presented as average ± SEM of typically three independent experiments (biological replicates).

### Modulation studies

For studies involving the modulation of plasma membrane potential, cells were incubated with ^99m^Tc-DMEOP or ^99m^Tc-TMEOP in a modified Earle’s medium (145 mM NaCl, 5.4 mM KCl, 1.2 mM CaCl_2_, 0.8 mM MgSO_4_, 0.8 mM NaH_2_PO_4_, 5.6 mM dextrose, 4.0 HEPES, and 1% FBS, pH 7.4 ± 0.1) or high K^+^ variants prepared by equimolar substitution of NaCl by potassium aspartate (to 60 and 120 mM, respectively). Additionally, a medium with high K^+^ concentration supplemented with valinomycin (1 µg/mL) was also tested. After 1 h of incubation, cell lysis was performed as described above. For mitochondrial membrane potential modulation, cells were incubated in normal culture medium with ^99m^Tc-DMEOP or ^99m^Tc-TMEOP in presence of Nigericin (5 µg/mL) and carbonylcyanide m-chlorophenylhydrazone (CCCP) (5 µM and 10 µM).

### Animal studies

The experiments were performed with female nude mice (BALBC nu/nu) at eight weeks of age (Charles River laboratories, CRIFFA, France). The animals were housed in a temperature- and humidity-controlled room with a 12 h light/12 h dark schedule. Furthermore, animals were kept in filter top cages, handled in laminar flow hoods, and maintained on sterilized diet and water ad libitum. All animal experiments were performed in compliance with Portuguese regulations for animal ethics and care.

#### Xenograft models

Cancer cells in serum-free media at a concentration with the desired inoculum (0.8-1 × 10^7^ cells) contained in 150 L were subcutaneously injected into the super scapular region of a group of nude mice. Only cell suspensions with viability greater than 95% were used. Mice were examined, weighed, and their tumors measured every two days. Tumors reached an appropriate size after approximately 2-4 weeks.

#### Biodistribution

Animals were intravenously injected into the tail vein with 100 µL of a solution of ^99m^Tc-TMEOP (0.5-1 MBq) in PBS pH 7.2 and were sacrificed by excess anesthesia at 1 and 4 h after injection. The injected activity (IA) was assumed to be the difference between the measured radioactivity in a dose calibrator (Curiemeter IGC-3, Aloka, Tokyo, Japan) in the syringe before and after injection. The organs of interest were dissected, rinsed with saline to remove excess blood, weighed, and their radioactivity was measured using a gamma-counter (LB2111, Berthold, Germany). The uptake in the tissues of interest was calculated and expressed as a percentage of the injected radioactivity per gram of tissue (% IA/g). For blood, bone, and muscle, total activity was estimated assuming that these organs constitute 6%, 10%, and 40% of the total body weight, respectively. Statistical analysis of the data (*t*-test) was done with GraphPad Prism and the level of significance was set as 0.05.

### Western blot

Cells were lysed in Cell Lytic-MT Extraction reagent (Sigma) supplemented with Complete protease inhibitor cocktail tablets (Roche Applied Science). After 15 min on ice, lysates were centrifuged at 14,000 *g* for 10 min at 4 °C to pellet the cellular debris and the supernatants were removed for further use. The total protein content was determined by using the DC Protein Assay Kit (Biorad) and aliquots of protein (100 μg) from each sample were analyzed using standard Western blot procedures. Briefly, protein extracts were subjected to electrophoresis on a 7% sodium dodecyl sulfate-polyacrylamide gel and transferred electrophoretically onto nitrocellulose membranes. The blots were blocked with PBS-tween (PBS-T) containing 5% nonfat dry milk for 1 h. Then, the blotting membranes were incubated with primary antibodies against Pgp (1:1000, Abcam), MRP1 (1:500, Abcam), and actin (1:8000, Sigma) overnight. Membranes were washed with PBS-T and incubated for 1 h with secondary antibody (goat anti-mouse IgG-HRP, Biorad) diluted 1:3000. Finally, membranes were developed using the SuperSignal WestPico Substrate kit (Pierce, Rockford, IL) according to the manufacturer’s instructions. Samples of the xenotransplants were excised and mechanically fragmented. Then, the samples were lysed and processed as described above.

## Results

As previously reported, the cationic lipophilic complexes ^99m^Tc-DMEOP and ^99m^Tc-TMEOP could be obtained in quantitative yield and with high radiochemical purity (> 99%), being used in the biological studies reported herein without any purification^[26]^.

### *In vitro* evaluation of tumoral detection

#### Uptake in tumoral cells

**Figure 2:**
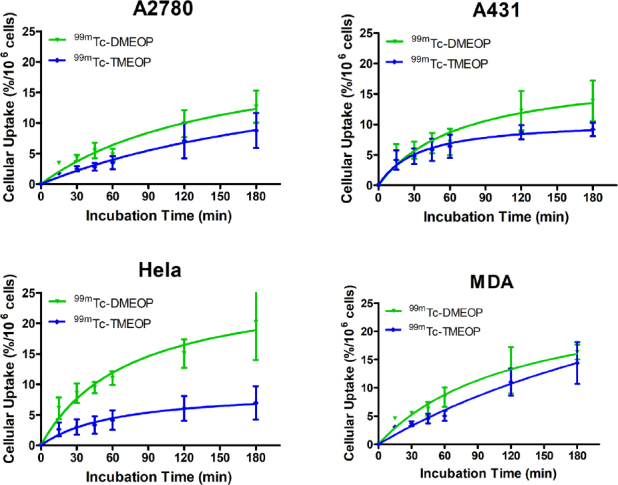
Cellular uptake of ^99m^Tc-DMEOP and ^99m^Tc-TMEOP in several human cancer cell lines, represented as the percentage of cell-associated radioactivity normalized to 10^6^ cells over time. The results presented were calculated from independent biological replicates (*n* = 3) and are given as the average ± SEM

Cellular uptake assays were firstly conducted to evaluate the ability of the two radiocomplexes to enter into tumoral cells. A panel of cellular models from different types of human tumors was used, in order to get an insight into the biological selectivity of the complexes, and, afterwards, for some selected cases, a comparison with radiopharmaceuticals in clinical use, ^99m^Tc-Sestamibi and ^99m^Tc-tetrofosmin, was also performed. Figure 2 presents the results for the uptake kinetics in ovarian A2780, vulvar A431, cervix Hela and breast MDA MB231 carcinoma cell lines. The results show that cellular uptake increased as a function of incubation time for all tumoral cell lines, with ^99m^Tc-DMEOP generally presenting higher uptake values than ^99m^Tc-TMEOP. After 3 h of incubation, Hela cells exhibited the highest uptake of ^99m^Tc-DMEOP (with almost 20% of applied activity associated with the cells), followed by MDA MB231 (with almost 18%) and then A2780 and A431 with similar values (between 12% and 13%). In Hela, A2780, and A431 cell lines, ^99m^Tc-TMEOP presented uptake values around 10% (7%, 8%, and 9% of applied activity associated with the cells, respectively) and, in MDA MB231 cell line, the uptake value was more similar to the one presented by ^99m^Tc-DMEOP (14% and 18%, respectively).

**Figure 3:**
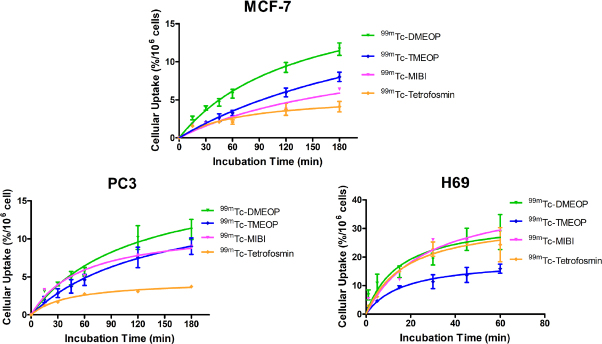
Cellular uptake of ^99m^Tc-DMEOP, ^99m^Tc-TMEOP, ^99m^Tc-Sestamibi, and ^99m^Tc-tetrofosmin in several human cancer cell lines, represented as the percentage of cell-associated radioactivity normalized to 10^6^ cells over time. The results presented were calculated from independent biological replicates (*n* = 3-8) and are given as the average ± SEM

Next, we evaluated the uptake of our two radiocomplexes in comparison with the radiopharmaceuticals in clinical use, ^99m^Tc-Sestamibi and ^99m^Tc-tetrofosmin, which have been shown to accumulate in tumoral cells *in vitro* and *in vivo*. For this study, we considered the MCF7, H69, and PC3 cancer cell lines [Figure 3]. We selected these cell lines because breast, lung, and prostate cancers are among the ones that more often develop resistance to treatment as well as present high metastatic potential.

In the breast cancer cell line MCF7 and in the prostate cancer cell line PC3, ^99m^Tc-DMEOP and ^99m^Tc-TMEOP presented a moderate uptake with values between 8% and 9% at 3 h for ^99m^Tc-TMEOP and slightly above 11%-12% at the same time point for ^99m^Tc-DMEOP. In these cell lines, ^99m^Tc-MIBI behaved similarly to ^99m^Tc-DMEOP. This is in contrast with ^99m^Tc-tetrofosmin, which presents the lowest uptake values (3% and 4% at 3 h for PC3 and MCF7, respectively). The small cell lung cancer H69 presented in general higher uptake values. This can be explained by the fact that, since it is a suspension cell line, the cell membrane area in contact with the radioactive medium is increased when compared with adherent monolayers. In this cell line, ^99m^Tc-DMEOP, ^99m^Tc-Sestamibi, and ^99m^Tc-tetrofosmin presented similar kinetics and uptake values at 1 h (28% of cell associated activity for ^99m^Tc-DMEOP and ^99m^Tc-Sestamibi and 24% for ^99m^Tc-tetrofosmin), with ^99m^Tc-TMEOP reaching values of ~60% of the uptake of the other compounds.

**Figure 4:**
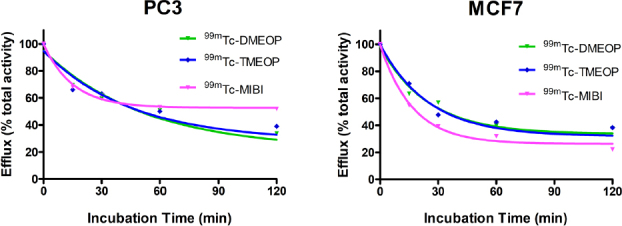
Cellular efflux of ^99m^Tc-DMEOP, ^99m^Tc-TMEOP, and ^99m^Tc-Sestamibi in several human cancer cell lines, represented as the percentage of cellular retention over a period of 2 h. The results presented were calculated from independent biological replicates (*n* = 3) and are given as the average ± SEM

To examine if ^99m^Tc-DMEOP, ^99m^Tc-TMEOP, and ^99m^Tc-Sestamibi might also exhibit similar profiles of intracellular retention in the adherent MCF7 and PC3 tumoral cell lines, we performed efflux experiments. The results obtained for the kinetics of efflux, after a loading incubation of 1 h, are presented in Figure 4. We observed a continuous and moderate washout for the three radiocomplexes throughout time in the two cell lines. For the PC3 cell line, ^99m^Tc-Sestamibi presented a slightly less pronounced efflux.

#### Mechanism of cellular uptake

In our initial characterization of the mechanism of myocardial uptake and subcellular localization of ^99m^Tc-TMEOP, we showed that this uptake is influenced by both the lipophilicity and the charge^[27]^. As hypothesized for other radiotracers^[29]^, the lipophilicity modulates the entry into the cellular and mitochondrial membranes, while the negative mitochondrial potential provides the electrochemical driving force for the radiotracers to localize in the myocyte’s mitochondrial compartments. *in vivo*, ^99m^Tc-TMEOP (and ^99m^Tc-DMEOP) tends to localize into the heart tissue where there is an increased mitochondrial population compared to other tissues. In most tumoral cells, the negative mitochondrial potential is greatly enhanced (approximately - 220 mV in cancer cells compared to approximately - 140 mV in normal cells)^[30,31]^, and therefore we tested if this could be the driving force for the uptake of our radiocomplexes in tumoral cells. To establish the influence of the plasma membrane potential (Δψp) and mitochondrial membrane potential (Δψm) on the uptake of these two radiocomplexes, MCF7 cells were incubated in different conditions that promoted the alteration or collapse of Δψp and/or Δψm.

**Figure 5:**
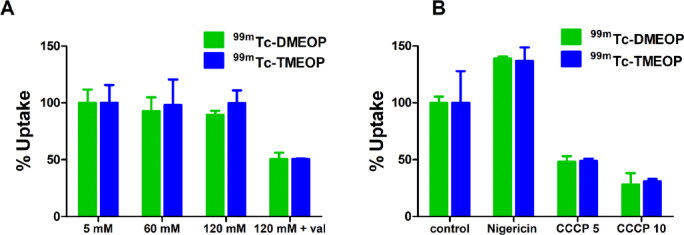
Cellular uptake of ^99m^Tc-DMEOP and ^99m^Tc-TMEOP in MCF7 cells incubated for 1 h with: (A) increasing concentrations of K^+^ and in the presence of valinomycin (1 µg/mL), where the results are presented as the percentage of uptake in relation to the medium with 5 mM K^+^; and (B) normal medium supplemented with nigericin (5 µg/mL) and CCCP (5 and 10 µM), where the results are presented as the percentage of uptake in relation to the normal medium. The results were calculated from independent biological replicates (*n* = 3) and are given as the average ± SEM. CCCP: carbonylcyanide m-chlorophenylhydrazone

Δψp is generated mainly by the movement of K^+^ ions from inside to outside the cell through K^+^ channels^[32,33]^. Therefore, to modulate the Δψp, experiments were performed in the presence of a buffer solution with increasing concentrations of K^+^ (5, 60, and 120 mM). The increase in K^+^ concentration in the medium from the physiological 5 mM to near 120 mM establishes an equilibrium between the extracellular and intracellular concentration of this ion, and as a direct consequence to depolarization of the plasma membrane. Under these experimental conditions, there is no expected influence of the Δψp in the cellular uptake of the radiotracers and their uptake can be exclusively related to Δψm. MCF7 cells were incubated in 5, 60, and 120 mM K^+^ modified media in the presence of ^99m^Tc-DMEOP and ^99m^Tc-TMEOP for 1 h. Moreover, an additional incubation with 120 mM K^+^ modified media in presence of valinomycin was also performed. Valinomycin is a potassium ionophore that increases membrane permeability to K^+^. Since Δψp is nearly zero for high extracellular concentrations of K^+^, the addition of valinomycin can lead to the collapse of Δψm due to the uncoupling of electron chain in oxidative phosphorylation^[30]^. The results of these experiments are presented in Figure 5A.

Modulation of Δψp through increasing extracellular K^+^ concentration did not affect the uptake of either compound, as the values obtained for each experimental condition are similar. On the other hand, upon the collapse of Δψm with valinomycin, there was a marked reduction of 50% on the cellular uptake of both ^99m^Tc-DMEOP and ^99m^Tc-TMEOP, confirming that Δψm is a critical factor for the intracellular accumulation of these compounds. To further investigate the roles of Δψp and Δψm in the uptake of ^99m^Tc-DMEOP and ^99m^Tc-TMEOP, incubations were performed in the presence of another set of ionophores: nigericin and CCCP. Nigericin mediates the electroneutral substitution of H^+^ for K^+^, in effect collapsing the pH gradient of the inner mitochondrial membrane with its subsequent hyperpolarization^[34]^. CCCP is a well known protonophore. It mediates the diffusion of H^+^ through the membranes and, as such, promotes the decoupling of pH gradient across the inner mitochondrial membrane, effectively collapsing Δψm selectively^[35]^. Upon MCF7 incubation in the presence of these compounds for 1 h, nigericin produced a 37% increase in the uptake for both ^99m^Tc-DMEOP and ^99m^Tc-TMEOP when compared with normal media [Figure 5B]. This result reflects, and is in accordance with, the expected hyperpolarization of the mitochondrial membrane. When cells were incubated with the radiocomplexes in the presence of CCCP, a dose-dependent reduction of uptake of 52%-72% for ^99m^Tc-DMEOP and 52%-70% for ^99m^Tc-TMEOP was observed, supporting the idea that Δψm is indeed the determinant driving force for the intracellular diffusion of these complexes.

### *In vitro* monitoring of multidrug resistance

**Figure 6:**
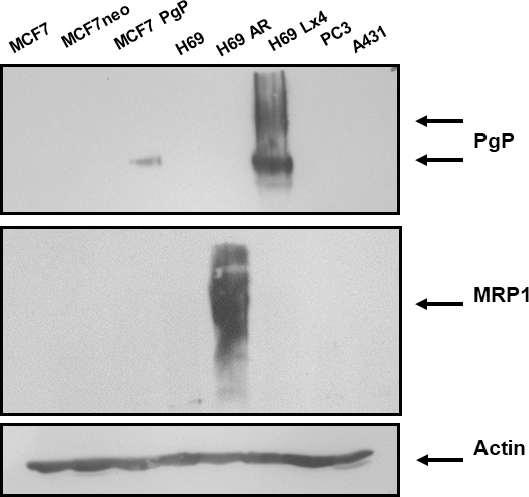
Expression of multidrug resistance proteins in human cancer cell lines. Western blot analysis was used to examine the protein expression levels of Pgp and MRP1. Actin was used as a loading control. MCF7 neo cells are a transfection control for MCF7 Pgp. Pgp: P-glycoprotein; MRP1: multidrug-resistant protein 1

After the evaluation of the performance of ^99m^Tc-DMEOP and ^99m^Tc-TMEOP for tumoral detection, the next step was the characterization of their ability to functionally assess MDR. We selected drug-resistant variants of the previously tested MCF7 breast and H69 lung cancer cell lines: MCF7 Pgp and H69 Lx4, which overexpress Pgp, and H69 AR, which overexpresses MRP1. We could confirm by Western blot the expression of these transporters in the cell lines selected, as well as their absence from the drug-sensitive cell lines [Figure 6].

**Figure 7:**
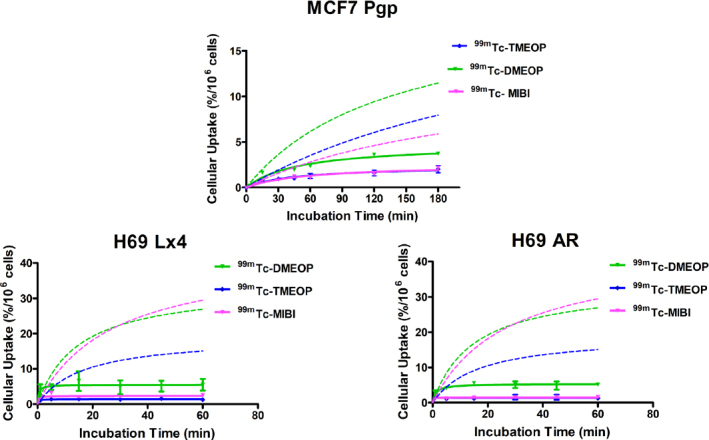
Cellular uptake of ^99m^Tc-DMEOP, ^99m^Tc-TMEOP, and ^99m^Tc-Sestamibi in several human drug resistant cancer cell lines: MCF7 Pgp and H69 Lx4 overexpressing the Pgp protein and H69 AR overexpressing MRP1. For comparison, the uptake curves in the corresponding non-resistant cell lines, MCF7 and H69 (discontinuous lines), are also represented. The results are presented as the percentage of cell-associated radioactivity normalized to 10^6^ cells over time, were calculated from independent biological replicates (*n* = 3-4), and are given as the average ± SEM. Pgp: P-glycoprotein; MRP1: multidrug-resistant protein 1

The uptake kinetics of ^99m^Tc-DMEOP and ^99m^Tc-TMEOP in comparison with ^99m^Tc-MIBI in the three cell lines tested are presented in Figure 7. For all compounds evaluated, the uptake in the drug resistant cell lines was greatly reduced when compared with the uptake in the non-resistant lines, which is plotted for comparison. For the MCF7 Pgp cell line, the values of uptake after 3 h incubation decreased 3.2 times for ^99m^Tc-DMEOP and ^99m^Tc-MIBI in comparison with MCF7 (from 12% to 3.7% and from 6.4% to 2% for ^99m^Tc-DMEOP and ^99m^Tc-MIBI, respectively), and almost 4.5 times for ^99m^Tc-TMEOP (from 8%-9% to 2%).

For the small cell lung carcinoma cell lines, a similar trend was observed: a reduction of the uptake values in the Pgp-overexpressing cell line H69 Lx4 compared with H69. The greatest reduction was observed for ^99m^Tc-TMEOP, which presents uptake values at 1 h more than 13 times inferior in the drug-resistant cells (from 15.9% to 1.2%). For ^99m^Tc-MIBI, the reduction of uptake in relation to the non-resistant H69 cell line was ~11 times (from 29% to 2.6% at 1 h) and for ^99m^Tc-DMEOP ~5 times (from 28.7% to 5.5% at 1 h). Interestingly, in the derivative line overexpressing MRP1, H69AR, the uptake of ^99m^Tc-TMEOP (and ^99m^Tc-MIBI) was almost null, being drastically reduced compared with the parental H69. This seems to suggest that this complex functions as a substrate of both Pgp and MRP1.

These results suggest that ^99m^Tc-TMEOP, despite having a slightly lower uptake in the cancer tumoral cells when compared with ^99m^Tc-DMEOP and in some cases with ^99m^Tc-MIBI (see Figure 3), presents the advantage of having more reduced uptake in the drug-resistant cell lines. ^99m^Tc-TMEOP is, from our two radiocomplexes studied, the one with the best ability to detect the MDR phenotype, as it seems to be a better substrate for Pgp and MRP1 than ^99m^Tc-DMEOP.

### Effect of a MDR modulator

**Figure 8:**
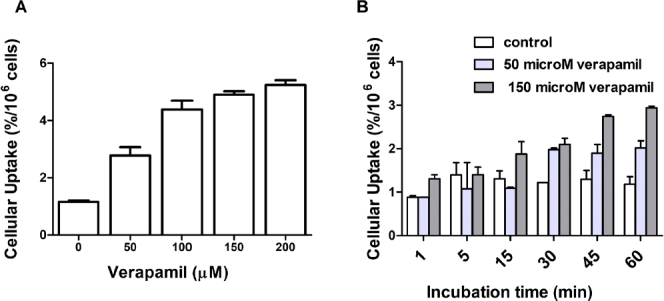
Cellular uptake of ^99m^Tc-TMEOP in H69 Lx4 cells in the presence of a Pgp modulator: (A) uptake at 1 h after incubation with increasing concentrations of verapamil; and (B) uptake over time after incubation with 50 and 150 M of verapamil. The results are presented as the percentage of cell-associated radioactivity normalized to 10^6^ cells, were calculated from independent biological replicates (*n* = 2), and are given as the average ± SEM. Pgp: P-glycoprotein

Based on the promising uptake results in the drug-resistant cell lines, and to confirm if the lower uptake in these resistant cell lines could be attributed to the overexpression of ABC transporters, we next studied the uptake of ^99m^Tc-TMEOP in the H69 Lx4 resistant cells in the presence of verapamil, a modulator of Pgp. To select the best concentration range of verapamil, a first screening was done using different concentrations at 1 h of incubation [Figure 8A].

**Figure 9:**
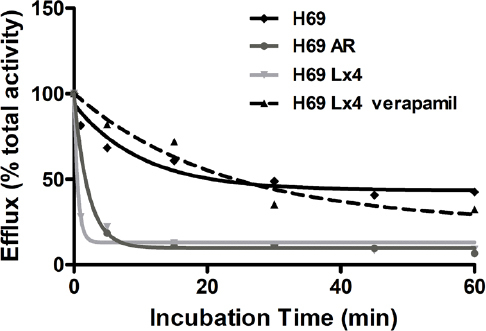
Cellular efflux of ^99m^Tc-TMEOP in H69 and drug-resistant variants H69 Lx4 and H69 AR and in H69 Lx4 incubated with 150 M of verapamil, presented as the percentage of cellular retention over a period of 1 h. The results presented were calculated from independent biological replicates (*n* = 2)

As can be seen in Figure 8A, there is a concentration-dependent increase of the cell uptake, compared to the control. We then selected 50 and 150 mM of verapamil to perform a time-dependent uptake assay [Figure 8B], which showed a time-dependent uptake of ^99m^Tc-TMEOP in the modulator-treated cells, in clear contrast with the non-treated cells. Additionally, we performed efflux studies in the presence of verapamil, as presented in Figure 9. The efflux of ^99m^Tc-TMEOP in H69 Lx4 and H69 AR is fast, with only ~20% of the radiocomplexes remaining in the cells at the 5-min time point. In the parental line, H69, which does not overexpress Pgp or MRP1, the efflux is lower and at a slower rate, with ~68% of intracellular ^99m^Tc-TMEOP at 5 min. Interestingly, when H69 Lx4 cells were incubated with verapamil, there was a clear reduction of the efflux of ^99m^Tc-TMEOP, to levels similar to the ones presented in H69 cells.

Taken together, the results of uptake and efflux in cancer cell lines overexpressing MDR-relevant proteins (Pgp and MRP1) confirm the potential of ^99m^Tc-DMEOP but more so of ^99m^Tc-TMEOP for the functional imaging of tumoral MDR. Therefore, we decided to study the *in vivo* behavior of the later in animal models with human cancer xenografts.

### *In vivo* studies

**Figure 10:**
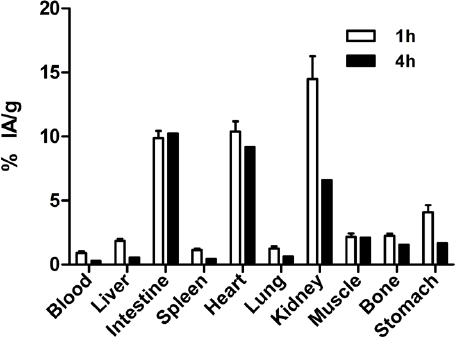
Biodistribution of ^99m^Tc-TMEOP in nude mice. Mice were injected intravenously with 0.6 MBq and sacrificed 1 and 4 h post-injection. The results are presented as the average ± SEM of the percentage IA/g tissue at each time point (*n* = 4). IA: injected activity

To evaluate *in vivo* the ability of ^99m^Tc-TMEOP for tumoral detection and imaging of MDR, we performed preliminary studies with nude mice with breast cancer xenografts. MCF7 and MCF7 Pgp cells were inoculated in nude mice and, when the tumor xenografts reached substantial size, biodistribution assays were carried out. Figure 10 presents the general biodistribution profile of ^99m^Tc-TMEOP in *BALB/c Nude* mice, which, in accordance with our previous results, presented a high and persistent heart uptake [~10% of injected activity/g tissue (% IA/g) at 1 h and 9.2 % IA/g at 4 h], with fast blood and liver clearance. A pronounced hepatobiliary excretion was found, as expected due to its lipophilic character; however, a relevant fraction was also eliminated through the kidney (~14.5 % IA/g at 2 h and 6.6 % IA/g at 4 h).

**Figure 11:**
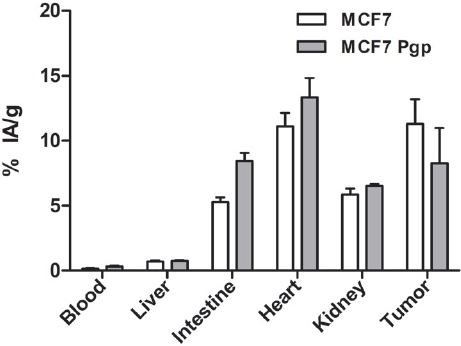
Biodistribution of ^99m^Tc-TMEOP in tumor-bearing nude mice with uptake in selected organs. Mice were injected intravenously with 0.6-1 MBq, and sacrificed at 4 h post-injection. The results are presented as the average ± SEM of the percentage injected activity/g tissue at each time point (*n* = 3-4). Pgp: P-glycoprotein

In tumor-bearing nude mice, the overall biodistribution profile of ^99m^Tc-TMEOP at 4 h post-injection was similar to non-tumor-bearing nude mice, as can been seen in Table 1 and Figure 11, where the uptake in the most relevant organs is presented.

**Table 1:** Biodistribution of ^99m^Tc-TMEOP in tumor-bearing nude mice

% IA/g	MCF7		MCF7 Pgp	
	Average	SEM	Average	SEM
Blood	0.17	0.02	0.33	0.04
Liver	0.73	0.08	0.75	0.06
Intestine	6.21	0.97	8.44	0.62
Spleen	0.72	0.11	0.58	0.08
Heart	10.75	0.94	13.33	1.50
Lung	0.81	0.07	1.02	0.09
Kidney	5.78	0.38	6.53	0.12
Muscle	2.54	0.24	2.69	0.14
Bone	1.86	0.18	2.15	0.31
Stomach	1.50	0.27	2.25	0.16
Tumor	11.30	1.89	8.26	2.72
Ratio tumor/blood	100.81	56.21	27.73	18.03
Ratio tumor/muscle	4.06	0.40	3.19	1.89

Mice were injected intravenously with 0.6-1 MBq and sacrificed at 4 h post-injection. The results are presented as the average ± SEM of the the percentage IA/g tissue at each time point (*n* = 3-4). Pgp: P-glycoprotein; IA: injected activity

In terms of tumoral uptake, an accumulation of ^99m^Tc-TMEOP in the xenografts was observed, with MCF7 xenografts presenting a higher uptake of ^99m^Tc-TMEOP when compared with MCF7 Pgp xenografts (although not statistically different). When considering the tumor/blood ratio, which at 4 h clearly showed the impact of blood clearance of the radioprobe, the animals with MCF7 xenografts presented a higher ratio than the ones with MCF7 Pgp tumors.

**Figure 12:**
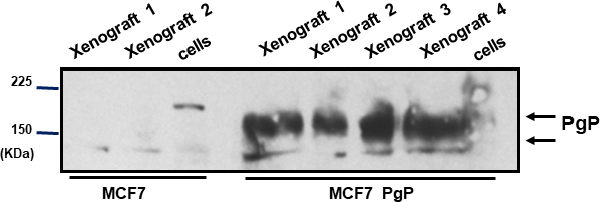
Expression of multidrug resistance proteins in human cancer cell lines and in tumor xenografts. Western blot analysis was used to examine the protein expression levels of the Pgp in the cell samples used to inoculate the mice and the xenografts developed. Pgp: P-glycoprotein

The biochemical characterization of the tumor xenografts was performed after the biodistribution assay, to confirm the expression of Pgp, as studies have showed that, in some situations, there is an alteration of the expression of Pgp in xenografts^[24]^. In parallel to the tumors xenografts, we also analyzed samples of the cell suspension used to inoculate the animals. As can be seen in Figure 12, neither the MCF7 cells nor the xenografts presented Pgp expression, and, conversely, both MCF7 Pgp cells and respective xenografts had high levels of Pgp expression. This supports the idea that the difference in tumoral uptake of ^99m^Tc-TMEOP observed *in vivo* is likely to be due to the efflux of the radiotracer by Pgp.

## Discussion

^99m^Tc-radiopharmaceuticals such as ^99m^Tc-sestamibi and ^99m^Tc-tetrofosmin are widely applied as myocardial blood flow tracers for clinical purposes; however, their high liver uptake can interfere in the analysis of cardiac imaging, mainly in the inferior left ventricular wall^[36-40]^. Our group has developed two ^99m^Tc complexes, ^99m^Tc-TMEOP and ^99m^Tc-DMEOP, with an improved biological profile in comparison with the radiotracers in clinical use, and demonstrated that ^99m^Tc-TMEOP accumulates intracellularly in mitochondria, and that its rapid liver clearance in mice can be explained by its transport by Pgp. These results, combined with the knowledge that similar cationic and lipophilic heart imaging agents have been explored for cancer detection and monitoring of tumor MDR function, led us to investigate the potential of these complexes for tumoral detection and MDR assessment *in vivo*.

Most tumoral cells present alterations in energy metabolism, increased transmembrane potential, and elevated reactive oxygen species generation, which can be explored as phenotypical traits for tumor targeting. In this context, we showed that there is a time-dependent uptake of ^99m^Tc-DMEOP and ^99m^Tc-TMEOP in human tumoral cell lines of different origins - lung, breast, prostate, cervix, ovary, and skin. For cell lines from breast, lung, and prostate cancer and under the same experimental conditions, we showed that these two complexes have similar uptake and efflux profiles as ^99m^Tc-Sestamibi, and are typically more concentrated in cancer cells than ^99m^Tc-tetrofosmin (except for H69 cell line). ^99m^Tc-DMEOP and ^99m^Tc-TMEOP have similar moderate lipophilicity values (log *P* values of 0.58 and 0.61, respectively), which are approximately half of the value for ^99m^Tc-Sestamibi (log *P* of 1.29). ^99m^Tc-tetrofosmin presents the lowest lipophilicity among the four tested radiocomplexes, which might explain its overall lower cell uptake^[41]^. As already demonstrated for ^99m^Tc-Sestamibi and other lipophilic cations, we showed that the cellular uptake of ^99m^Tc-DMEOP and ^99m^Tc-TMEOP is dependent of the mitochondrial membrane potential.

We then focused on the biological evaluation of these complexes in cancer cells lines with a MDR phenotype. Interestingly, we could show that ^99m^Tc-DMEOP and ^99m^Tc-TMEOP presented a reduced uptake in cancer cells lines expressing Pgp or MRP1, similarly to ^99m^Tc-Sestamibi. For ^99m^Tc-TMEOP, which seems to be a better substrate for Pgp and MRP1 than ^99m^Tc-DMEOP, we also demonstrated that, in the presence of a Pgp modulator, there is an increased uptake in the Pgp-expressing cancer cell line.

These encouraging results prompted a preliminary *in vivo* biological evaluation of ^99m^Tc-TMEOP in nude mice with human cancer xenografts. We selected breast cancer cell lines, negative and positive for Pgp expression, and performed biodistribution studies. The *in vivo* results show that, in the mice model selected, as expected, there is a typical high and persistent heart uptake, with rapid liver clearance most likely via Pgp expressed in these tissues. The uptake of ^99m^Tc-TMEOP in breast cancer xenografts is moderate to high with values of injected activity of ~11% at 4 h. When compared with the animals with the MDR tumor xenografts, the non-MDR xenografts present a higher uptake, although the difference is not statistically significant.

Taken together with the biochemical evidence of Pgp expression, these results seem to support that ^99m^Tc-TMEOP is a substrate of Pgp *in vivo*. Further studies with tumor xenografts of different cancer types and with Pgp and MRP1 modulators will help to clarify the potential of ^99m^Tc-TMEOP (and possibly ^99m^Tc-DMEOP) for *in vivo* non-invasive tumoral detection and MDR assessment.

## Declarations

### Copyright

© The Author(s) 2020.
